# Altered T cell phenotypes associated with clinical relapse of multiple sclerosis patients receiving fingolimod therapy

**DOI:** 10.1038/srep35314

**Published:** 2016-10-18

**Authors:** Chihiro Fujii, Takayuki Kondo, Hirofumi Ochi, Yoichiro Okada, Yuichiro Hashi, Tetsuya Adachi, Masaharu Shin-Ya, Sadayuki Matsumoto, Ryosuke Takahashi, Masanori Nakagawa, Toshiki Mizuno

**Affiliations:** 1 Department of Neurology, Graduate School of Medical Science, Kyoto Prefectural University of Medicine, Kyoto, Japan; 2 Department of Clinical Network and Collaborative Medicine, Kyoto University Hospital, Kyoto, Japan; 3 Department of Neurology, Kyoto University Graduate School of Medicine, Kyoto, Japan; 4 Department of Neurology, Tazuke Kofukai Foundation, Medical Research Institute, Kitano Hospital, Osaka, Japan; 5 Department of Neurology and Geriatric Medicine, Ehime University Graduate School of Medicine, Toon, Japan; 6 Department of Immunology, Kyoto Prefectural University of Medicine, Kyoto, Japan; 7 Department of Dental Medicine, Kyoto Prefectural University of Medicine, Kyoto, Japan; 8 Department of Neurology, North Medical Center, Kyoto Prefectural University of Medicine, Kyoto, Japan

## Abstract

Multiple sclerosis (MS) is a T cell-mediated autoimmune disease. Fingolimod, a highly effective disease-modifying drug for MS, retains CCR7^+^ central memory T cells in which autoaggressive T cells putatively exist, in secondary lymphoid organs, although relapse may still occur in some patients. Here, we analyzed the T cell phenotypes of fingolimod-treated, fingolimod-untreated patients, and healthy subjects. The frequency of CD56^+^ T cells and granzyme B-, perforin-, and Fas ligand-positive T cells significantly increased during fingolimod treatment. Each T cell subpopulation further increased during relapse. Interestingly, T cells from fingolimod-treated patients exhibited interferon-γ biased production, and more myelin basic protein-reactive cells was noted in CD56^+^ than in CD56^−^ T cells. It is likely that the altered T cell phenotypes play a role in MS relapse in fingolimod-treated patients. Further clinical studies are necessary to investigate whether altered T cell phenotypes are a biomarker for relapse under fingolimod therapy.

Multiple sclerosis (MS) is the most common demyelinating disease of the central nervous system (CNS) caused by T cell-mediated autoimmunity^1^. T helper (Th) 1 and Th17 cells are considered crucial in MS pathogenesis^1^,^2^. Before autoreactive Th1 and Th17 cells propagate the autoimmune response against self-antigens in the CNS, these cells undergo several important migration steps. The migration and distribution of T cells are largely controlled by adhesion molecules, chemokines, and their receptors^3^; thus, molecules related to immune cell migration are considered to be promising therapeutic targets for MS. To date, two agents that target the migration of immune cells have been approved for use in MS therapies. One of these is natalizumab, a recombinant humanized monoclonal antibody against the adhesion molecule α-4 integrin, which inhibits α-4 integrin-mediated adhesion of immune cells and interferes with their entry into the CNS^4^. The other is fingolimod, which causes aberrant internalization of sphingosine-1-phosphate 1 (S1P1) receptors and inhibits lymphocyte egress from secondary lymphoid organs (SLO)^5^. While fingolomod mainly influences the naïve T cells and central memory T cells (TCM) expressing the homing receptor CCR7 from SLO, it exerts little effect on CCR7^-^ effector memory T cells (TEM), which circulate throughout the body^6^,^7^,^8^,^9^.

Clinical trials have demonstrated the superior efficacy of fingolimod in reducing clinical relapses and magnetic resonance imaging activities in MS^10^,^11^, suggesting that encephalitogenic T cells are primarily TCM. This is consistent with a previous report showing that most cerebrospinal fluid (CSF) T cells are CCR7^+^ TCM in MS patients^12^. It has been proposed that an insufficient reduction in TCM in peripheral blood^13^ and the retention of TCM in CSF^14^ are associated with clinical relapses during fingolimod therapy. However, whether the frequency of TCM in relapsed patients is higher than that in relapse-free patients receiving fingolimod has not been confirmed. Thus, it is possible that relapse while receiving fingolimod has other underlying immunopathological mechanisms than the insufficient reduction of TCM. There is another observation to indicate distinct pathomechanisms of the therapy-associated relapse. It was reported that relapsed lesions during fingolimod therapy tended to be unusually severe or tumefactive^15^,^16^,^17^.

In this study, we investigated the phenotypic and functional characteristics of peripheral blood T cells in patients undergoing fingolimod therapy both in remission and at relapse cpmpared with fingolimod-untreated MS patients and healthy subjects. Our results showed that the T cell phenotypes were altered under fingolimod therapy, and that these altered T cell phenotypes were remarkably increased during relapse. Thus, we propose that altered T cell phenotypes are associated with relapse under fingolimod therapy.

## Results

### Fingolimod therapy increases the frequency of CD56^+^ T cells in peripheral blood

**Figure 1:**
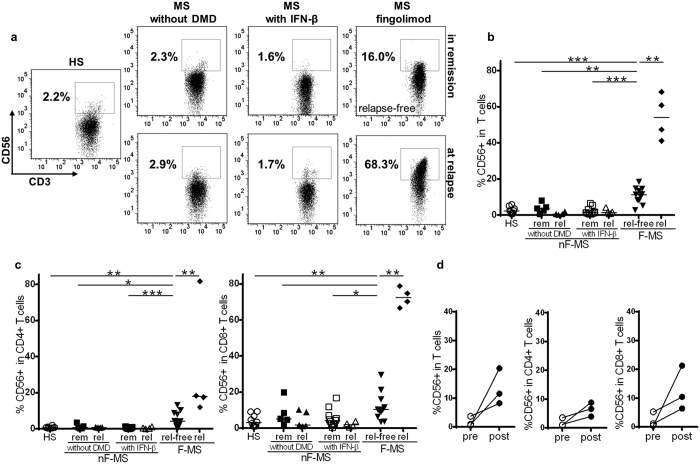
Frequency of CD56^+^ T cells in healthy subjects (HS) and patients with multiple sclerosis (MS). (**a**) CD56 expression on CD3-gated T cells. Representative FACS dot plots of HS, MS patients without disease-modifying drug (MS without DMD), MS patients with IFN-β therapy (MS with IFN-β), and those with fingolimod therapy (MS with fingolimod) are shown. (**b**) The frequency of CD56^+^ cells in CD3^+^ T cells in the indicated groups. (**c**) The frequency of CD56^+^ cells in CD4^+^ T cells in the indicated groups (left), and that of CD56^+^ cells in CD8^+^ T cells in the indicated groups (right). (**d**) The frequency of CD56^+^ T cells before and after fingolimod treatment in MS patients. The frequency of CD56^+^ T cells within total T cells, and CD4^+^ and CD8^+^ T cell subsets is shown. nF-MS: multiple sclerosis patients without fingolimod therapy, F-MS: multiple sclerosis patients with fingolimod therapy, rem: remission, rel: relapse, rel-free: relapse-free. Bars represent median values. p-values: *p < 0.05; **p < 0.01; ***p < 0.001.

We intensively studied surface molecules on peripheral blood (PB) T cells from fingolimod-treated MS (F-MS) patients, interferon (IFN)-β-treated patient, patients not treated with disease modifying dugs (DMD) and healthy subjects (HS) (Table 1). The data revealed that F-MS patients had a higher frequency of CD56^+^ T cells in peripheral blood T cells. CD56 expression on T cells was analyzed because previous reports have demonstrated a possible association of CD56^+^ T cells with pathogenesis of MS^18^,^19^. Relapse-free F-MS showed a significantly higher frequency of CD56^+^ T cells (the mean frequency, 10.8%) compared with HS (2.5%, p < 0.0001), IFN-β-treated patients (2.2%, p < 0.0001), and patients not treated with DMD (3.9%, p = 0.0055) in remission (Fig. 1a,b). This elevated CD56 expression was observed within both CD4^+^ and CD8^+^ T cell subsets (Fig. 1c). Moreover, the frequency of CD56^+^ T cells markedly increased in all of the four relapsed F-MS patients whose samples were available at relapse; the frequency of CD56^+^ T cells increased to 68.3%, 60.9%, 47.4%, and 41.2%. Thus, the relapsed F-MS group exhibited a significantly higher frequency of CD56^+^ T cells compared to the relapse-free F-MS group (the mean frequency, 54.4% *vs* 10.8%, p = 0.0015) (Fig. 1b). The upregulated frequency of CD56^+^ cells at relapse in the F-MS group was also observed in both the CD4^+^ and CD8^+^ T cell subsets (the mean frequency, CD56^+^CD4^+^ T cells: 32.4% *vs* 5.3% p = 0.0029; CD56^+^CD8^+^ T cells: 72.6% *vs* 12.0%, p = 0.0015) (Fig. 1c). In contrast to F-MS, both untreated and IFN-β-treated patients showed little difference in the frequency of CD56^+^ cells within total T cells and CD4^+^ and CD8^+^ T cell subsets, regardless of disease phase. Thus, we placed untreated and IFN-β-treated patients into the same group as non-fingolimod-treated patients (nF-MS) in this study because the frequency of CD56^+^ T cells was not affected by IFN-β therapy.

To confirm the influence of fingolimod therapy on CD56 expression, we analyzed the frequency of CD56^+^ cells within total T cells and CD4^+^ and CD8^+^ T cell subsets before and after the induction of fingolimod therapy (Fig. 1d). The frequency of CD56^+^ T cells increased after fingolimod therapy in all patients analyzed, and the average frequency of CD56^+^ T cells increased to 13.4% from 1.7%. CD56 positivity also increased to 6.4% from 1.9% in CD4^+^ T cells and to 12.7% from 2.3% in CD8^+^ T cells.

Absolute numbers of CD56^+^ T cells did not remarkably differ between nF-MS and F-MS in remission (Table 2). However, those of CD56^+^ T cells at relapse in F-MS patients were higher than those in nF-MS despite the remarkable decrease of total CD3^+^ T cells in F-MS.

### Frequency of CCR7^+^ T cells is not high during relapse on fingolimod, and CD56 expression is elevated regardless of CCR7 expression

**Figure 2:**
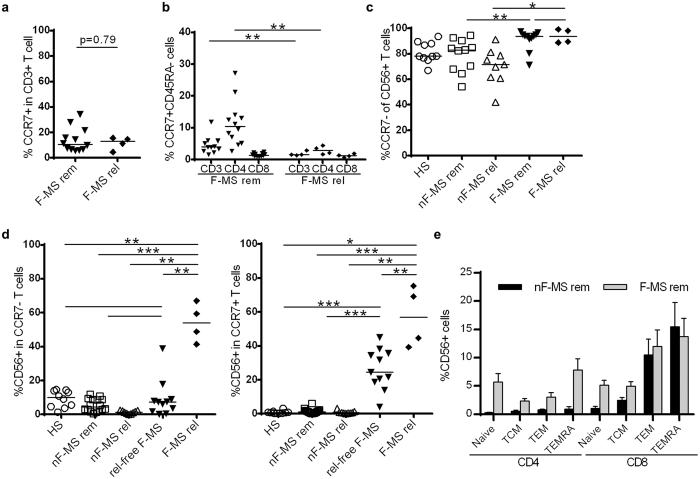
Association of CD56 and CCR7 expression on T cells in healthy subjects (HS) and multiple sclerosis (MS) patients. (**a**) The frequency of CCR7^+^ T cells within CD3^+^ T cells in F-MS patients both in remission and at relapse. (**b**) The frequency of central memory T cells (CCR7^+^CD45RA^−^ T cells) within CD3^+^, CD4^+^ or CD8^+^ T cells in F-MS patients both in remission and at relapse. (**c**) The frequency of CCR7^−^ T cells within CD56^+^ T cells in HS and each patient group. (**d**) The frequency of CD56^+^ cells within CCR7^−^ and CCR7^+^ T cells. (**e**) The frequency of CD56^+^ cells within naïve (CCR7^+^CD45RA^+^), central memory (CCR7^+^CD45RA^−^) (TCM), effector memory (CCR7^−^CD45RA^−^) (TEM) and CD45RA^+^ TEM (TEMRA) subsets in CD4^+^ and CD8^+^ T cells. nF-MS: multiple sclerosis patients without fingolimod therapy, F-MS: multiple sclerosis patients with fingolimod therapy, rem: remission, rel: relapse, rel-free: relapse-free. Bars represent median values. p-values: *p < 0.05; **p < 0.01; ***p < 0.001.

To investigate whether an insufficient reduction of TCM in peripheral blood is related to relapse in patients receiving fingolimod, we evaluated the frequency of CCR7^+^ T cells in peripheral blood. The frequency in peripheral blood from relapsed F-MS patients was not necessarily higher than that in relapse-free F-MS patients (Fig. 2a). The frequency of CCR7^+^ T cells was higher in four of the 12 relapse-free F-MS patients than the highest frequency among relapsed F-MS patients. Moreover, in contrast to previous reports^13^, the frequency of total TCM and of CD4^+^ TCM significantly decreased in relapsed F-MS patients compared to relapse-free F-MS patients (Fig. 2b). We further analyzed the association between the expression of CD56 and CCR7 because T cells expressing CCR7 are primarily affected by fingolimod. Most CD56^+^ T cells did not express CCR7 in all of the groups (Fig. 2c). Although relapse-free F-MS showed no significant difference in the frequency of CD56^+^ cells in CCR7^−^ T cells compared to in HS and nF-MS, the four relapsed F-MS patients showed a significantly higher frequency of CD56^+^ cells within the CCR7^−^ T cell population compared to all other groups (p < 0.003) (Fig. 2d, left). Notably, the frequency of CD56^+^ cells among CCR7^+^ T cells increased significantly even in relapse-free F-MS patients compared to in HS and nF-MS (p < 0.0001), and increased significantly in relapsed F-MS patients as well (the mean frequency, 26.2% n relapse-free F-MS *vs* 57.1% in relapsed F-MS, p = 0.0059) (Fig. 2d, right). CD56 expression on nearly every T cell subset, naïve (CCR7^+^CD45RA^+^), central memory (CCR7^+^CD45RA^−^) effector memory (CCR7^−^CD45RA^−^) and CD45RA+ effector memory (TEMRA) subsets in CD4^+^ and CD8^+^ T cells, increased in F-MS (Fig. 2e). Our results demonstrate that fingolimod therapy induced CD56 expression on T cells regardless of CCR7 expression or T cell subpopulations, particularly at relapse.

### Neither fingolimod nor fingolimod-phosphate directly induce CD56 expression on T cells *in vitro*

**Figure 3:**
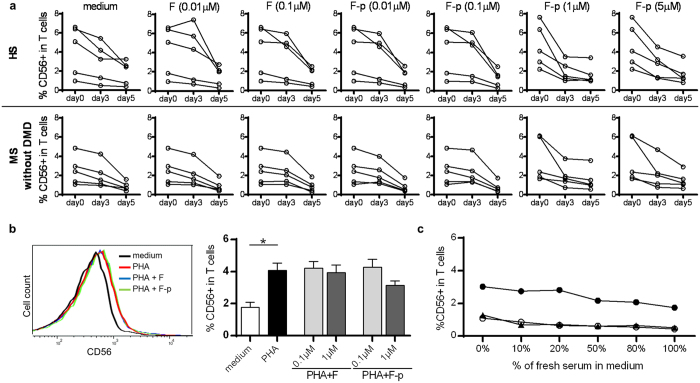
Effect of fingolimod, fingolimod-phosphate and the serums of patients under fingolimod therapy on CD56 expression *in vitro*. (**a**) Kinetic change of CD56^+^ T cells co-cultured with fingolimod (F) or fingolimod-phosphate (F-p) for 5 days in healthy subjects (HS) and multiple sclerosis patients without disease-modifying drug (MS without DMD). (**b**) CD56 expression on T cells stimulated by PHA in the presence or absence of fingolimod or fingolimod phosphate at a concentration of 0.1 and 1 μM. Representative graphs of CD56 expression on T cells cultured in the presence of PHA, PHA with 0.1 μM fingolimod and PHA with 0.1 μM fingolimod-phosphate (left panel) (**c**) CD56 expression on T cells of MS without DMD co-cultured with serum of the same patient after fingolimod therapy. Each column represents mean ± SEM. p-values: *p < 0.05.

To evaluate the direct effects of fingolimod and fingolimod phosphate on CD56 expression, peripheral blood mononuclear cells (PBMC) were derived from HS and MS patients without DMD, and were cultured with fingolimod or fingolimod-phosphate at the concentration of 0.01, 0.1, 1, 5, and 10 μM. The concentrations were determined to be higher than 0.0073 μM, which is the steady-state concentration of the blood of fingolimod-treated patients^20^. The frequency of CD56^+^ T cells rather decreased even in the presence of either fingolimod or fingolimod-phosphate (Fig. 3a). Fingolimod concentrations greater than 1 μM and fingolimod-phosphate at the concentration of 10 μM induced significant cell death. Next, we evaluated whether upregulation of CD56 on T cells induced by co-culture with phytohemagglutinin (PHA) further increase when fingolimod or fingolimod-phosphate was added to the culture. The presence of fingolimod or fingolimod-phosphate did not have any additional effect on CD56 expression (Fig. 3b).

### Serum from fingolimod treated patients does not induce CD56 upregulation

To study whether serum from fingolimod-treated patients can upregulate CD56 on T cells, we co-cultured PBMC unexposed to fingolimod with various densities of autologous serum. The serum was collected after the initiation of fingolimod therapy, and the frequency of CD56^+^ T cells was confirmed to be more than 10% of the total T cells at serum sampling. The experiments revealed that the frequency of CD56^+^ T cells did not increase in the presence of fingolimod-exposed serum (Fig. 3c).

### Fingolimod therapy upregulates the expression of cytotoxic molecules on T cells

**Figure 4:**
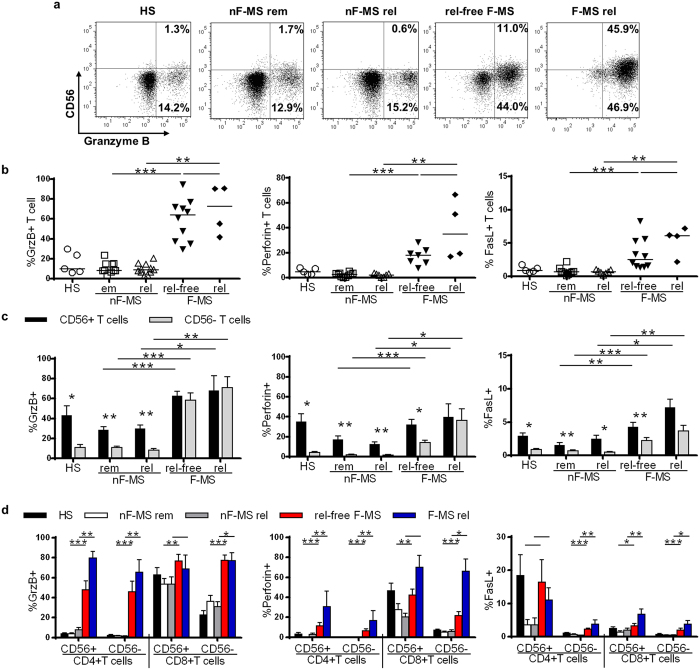
Expression of cytotoxic molecules in each T cell subset of healthy subjects (HS) and multiple sclerosis (MS) patients. (**a**) Representative analysis of CD56 and granzyme B expression, gated on CD3^+^ T cells from left to right: a healthy subject, an MS patient not on fingolimod in remission (nF-MS rem) and one at relapse (nF-MS rel), a relapse-free MS patient with fingolimod therapy (rel-free F-MS) and one at relapse (F-MS rel). (**b**) The expression of granzyme B (GrzB), perforin, and Fas-Ligand (FasL) in or on CD3^+^ T cells are shown. (**c**) The frequency of cells expressing each cytotoxic molecule was compared between CD56^+^ and CD56^−^ T cells. (**d**) The frequency of cells expressing each cytotoxic molecule in CD4^+^CD56^+^ T cells, CD4^+^CD56^−^ T cells, CD8^+^CD56^+^ T cells, and CD8^+^CD56^−^ T cells. Bars represent median values. Each column represents mean ± SEM. p-values: *p < 0.05; **p < 0.01; ***p < 0.001.

Because CD56 expression has been reported to be associated with T cell cytotoxicity^21^,^22^, we analyzed the expression of granzyme B, perforin, and Fas ligand (FasL) in or on T cells (Fig. 4). These three molecules were significantly upregulated in F-MS patients compared to in nF-MS and HS (nF-MS and HS, granzyme B: p < 0.0001 and p = 0.013; perforin: p = 0.0001 and p = 0.0051; FasL: p = 0.0002 and p = 0.0085). The relapse-free F-MS group showed increased frequencies of granzyme B-, perforin- and FasL-positive T cells, with average frequencies of 59.2%, 16.5% and 3.3%, respectively (Fig. 4a,b). Moreover, the relapsed F-MS patients showed further increases in the frequencies of these molecules, averaging 69.5%, 38.5% and 5.4%, respectively (Fig. 4a,b).

Next, we evaluated the expression of cytotoxic molecules in CD56^+^ and CD56^−^ T cells. Granzyme B-, perforin- and FasL-expressing T cells resided largely in the CD56^+^ fraction in both HS and nF-MS (Fig. 4c). In contrast, these cytotoxic molecule-expressing cells in F-MS patients increased within both the CD56^+^ and the CD56^−^ T cell subset. The frequency of CD56^−^ T cells expressing these cytotoxic molecules significantly increased in F-MS patients compared to in HS and nF-MS patients (p < 0.05 for each comparison). The highest frequencies of cytotoxic molecule-expressing CD56^+^ T cells and CD56^−^ T cells were observed in the relapsed F-MS patients.

It is generally regarded that the cytotoxic capability of T cells is predominantly attributed to CD8^+^ T cells. To evaluate this assumption, we further analyzed cells expressing cytotoxic molecules both in CD4^+^ and CD8^+^ T cells (Fig. 4d). CD56^+^CD8^+^ T cells commonly expressed granzyme B and perforin in all of the groups. However, the frequency of granzyme B- and perforin-expressing T cells in the F-MS group significantly increased not only in the CD56^+^CD8^+^ T cells subset but also in the CD4^+^ and CD56^−^ T cell subsets compared to other groups (Fig. 4d). The frequency of FasL-expressing T cells in the F-MS group was also elevated in all T cell subpopulations. These results indicate that fingolimod therapy induced the aberrant expression of cytotoxic molecules in or on T cells. The frequency of cytotoxic molecule-expressing T cell subpopulations did not differ between at relapse and in remission in nF-MS patients; however, they generally increased in relapsed F-MS patients compared to in relapse-free F-MS.

### Fingolimod therapy increases the frequency of IFN-γ-producing T cells upon phorbol 12-myristate 13-acetate (PMA)/ionomycin stimulation *in vitro*

**Figure 5:**
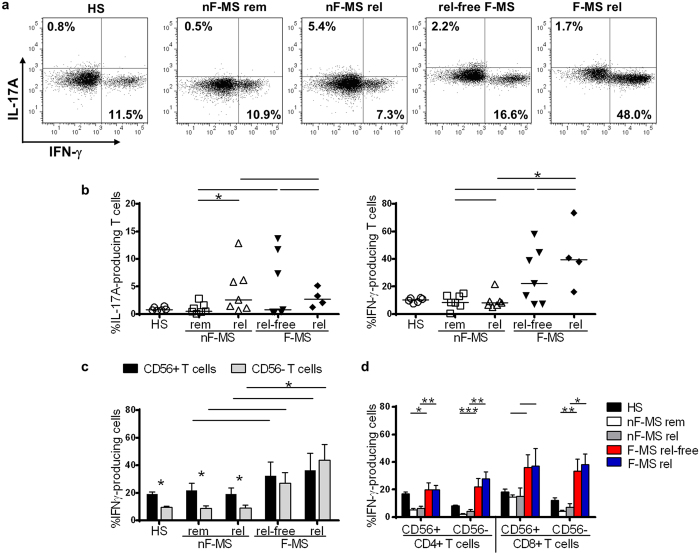
The frequency of proinflammatory cytokine-producing cells in response to PMA/ionomycin stimulation. (**a**) Representative data of FACS dot plots analyzing IFN-γ and IL-17A production gated on CD3^+^ T cells. Depicted as follows from left to right, a healthy subject (HS), a multiple sclerosis (MS) patient not on fingolimod in remission (nF-MS rem) and one at relapse (nF-MS rel), and a relapse-free patient with fingolimod therapy (rel-free F-MS) and one at relapse (F-MS rel). (**b**) The frequency of IL-17A-producing cells in CD3^+^ T cells (left) and that of IFN-γ-producing cells in CD3^+^ T cells (right). (**c**) The frequency of IFN-γ producing cells was compared between CD56^+^ and CD56^−^ T cells. (**d**) The frequency of IFN-γ-producing cells in CD4^+^CD56^+^ T cells and CD4^+^CD56^−^ T cells, CD8^+^CD56^+^ T cells and CD8^+^CD56^−^ T cells. Bars represent median values. Each column represents mean ± SEM. P-values: *p < 0.05; **p < 0.01; ***p < 0.001.

We analyzed the ability of T cells to produce IFN-γ and IL-17A in response to PMA/ionomycin stimulation *in vitro* (Fig. 5). There was no difference in the frequency of IL-17A-producing T cells between HS and nF-MS patients in remission; however, IL-17A-producing T cells increased significantly in nF-MS patients at relapse compared to in remission (the mean frequency, 4.4% *vs* 0.98%, p = 0.037) (Fig. 5a,b). In contrast to nF-MS patients, the frequency of IL-17A-producing T cells in F-MS did not significantly differ between the relapse-free and the relapsed patients. The mean frequency of IFN-γ-producing T cells in HS and nF-MS patients were similar (approximately 10%). However, F-MS patients showed an increased frequency of IFN-γ-producing T cells compared to in HS and nF-MS patients, and this tendency was greater in the relapsed F-MS than in the relapse-free F-MS patients (42.1% *vs* 27.4%).

Because we found a substantial increase in the frequency of IFN-γ-producing T cells in F-MS patients, we subsequently examined these cells in association with CD56 expression (Fig. 5c). CD56^+^ T cells were more likely to produce IFN-γ than CD56^−^ T cells in HS and nF-MS patients, regardless of the disease phase. In F-MS patients, the mean frequency of IFN-γ-producing cells in CD56^−^ T cells was increased both in relapse-free F-MS (27.1%) and relapsed F-MS (43.7%) patients compared with HS (9.7%) and nF-MS patients (8.7% in remission and 9.0% at relapse). The mean frequency of IFN-γ-producing cells both in CD56^+^ and CD56^−^ T cells was increased in relapsed F-MS patients compared to in relapse-free F-MS patients. This trend was consistent for both CD4^+^ and CD8^+^ T cell subpopulations (Fig. 5d).

### CD56^+^ T cells produce IFN-γ in response to myelin basic protein (MBP) in patients on fingolimod therapy

**Figure 6:**
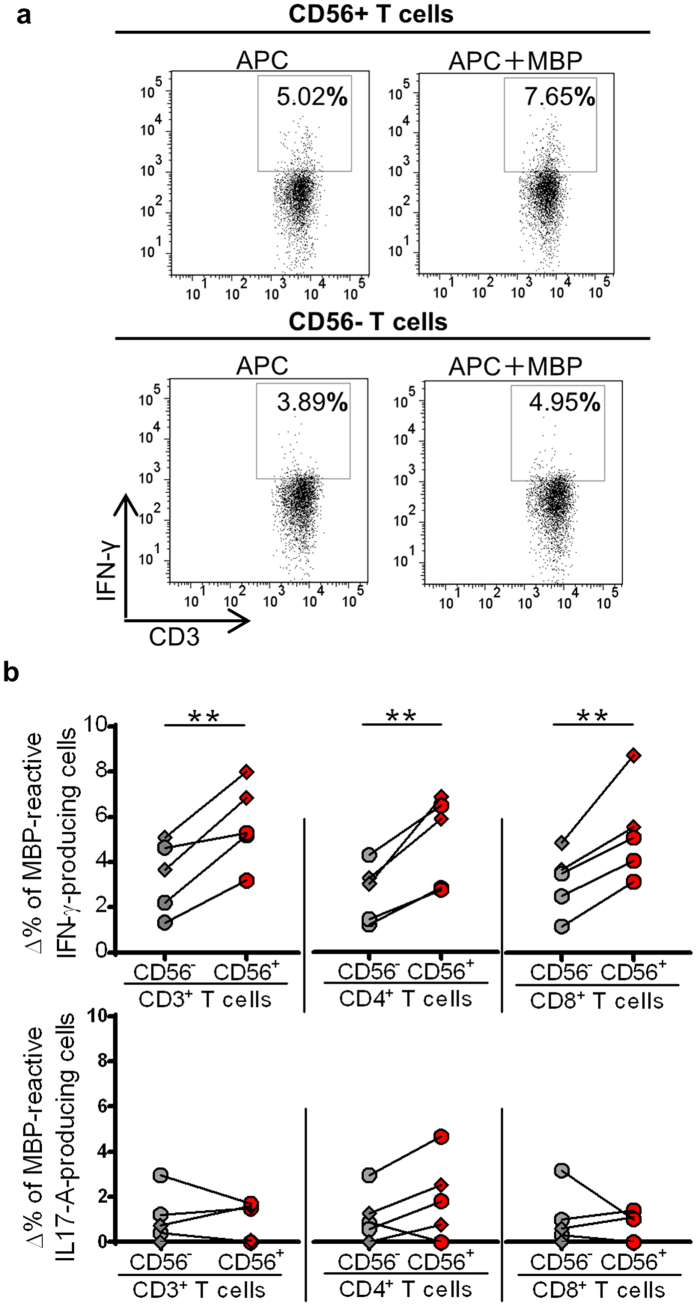
Myelin basic protein (MBP)-reactive proinflammatory cytokine production by CD56^+^ T cells from multiple sclerosis (MS) patients on fingolimod in remission. CD56^+^ and CD56^−^ T cells were co-cultured with antigen-presenting cells (APC) in the presence or absence of MBP peptide mixture, and intracellular cytokines were analyzed by flow cytometry. (**a**) Representative FACS dot plots of IFN-γ-producing cells in CD56^+^ and CD56^−^ T cells when the cells were co-cultured with APC in the presence or absence of MBP. (**b**) The delta frequency of cells that produced IFN-γ (upper row) and IL-17A (bottom row) within CD56^+^ and CD56^−^ T cells. The delta frequency was calculated by subtracting the frequency of cytokine-producing cells in the absence of MBP from that in the presence of MBP. Diamonds are data from relapse-experienced MS patients with fingolimod therapy in remission, and circles represent relapse-free MS patients while on fingolimod treatment. Bars represent median values. p-values: **p < 0.01.

Because both CD56^−^ and CD56^+^ T cells produced the cytotoxic molecules and IFN-γ in F-MS patients, it is important to understand whether these cells included autoreactive T cells. To address this question, we analyzed the reactivity of these cells against MBP, one of the well-studied autoantigens for MS (Fig. 6a). The delta frequency of IFN-γ-producing cells in response to MBP was significantly higher in CD56^+^ T cells than in CD56^−^ T cells (5.7% *vs* 3.4%, p = 0.0082), and this tendency was true in both CD4^+^ (5.0% *vs* 2.7%, p = 0.0066) and CD8^+^ T cells (5.3% *vs* 3.2%, p = 0.0072) (Fig. 6b, upper row). The change in the frequency of IL-17A-producing cells in response to MBP was much lower in both CD56^+^ and CD56^−^ T cells (0.9% *vs* 1.0%, p = 0.82), and this observation was consistent for both CD4^+^ (1.9% *vs* 1.1%, p = 0.13) and CD8^+^ T cells (0.7% *vs* 1.0%, p = 0.54) (Fig. 6b, bottom row). These results indicate that the increased CD56^+^ T cells in F-MS patients could produce IFN-γ in response to MBP.

### Longitudinal study of relapse-experienced patients on fingolimod therapy

**Figure 7:**
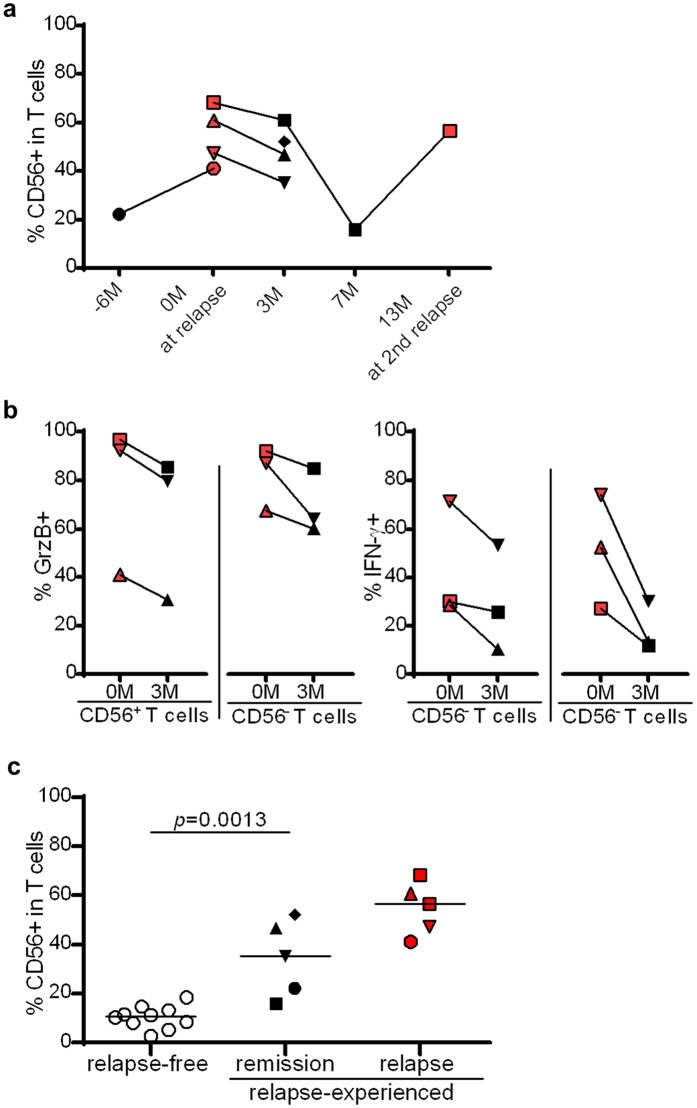
Longitudinal analysis of CD56 expression, granzyme B (GrzB) expression, and frequency of IFN-γ-producing cells in relapse-experienced multiple sclerosis (MS) patients on fingolimod. (**a**) Temporal changes in the frequency of CD56-expressing cells within the CD3^+^ T cell fraction. (**b**) The frequency of GrzB-expressing cells and IFN-γ-producing cells in CD56^+^ and CD56^−^ T cell subsets decreased 3 months after relapse. (**c**) The frequency of CD56 expression in relapse-experienced MS patients with fingolimod therapy in remission remained higher than in patients who did not have any relapse while on fingolimod treatment. Each relapse-experienced patient is indicated by different-shaped dots. The frequencies at relapse are presented as red dots, and those in remission are presented as black dots. Bars represent median values. p-values: **p < 0.01.

In this study, we analyzed five relapse-experienced F-MS patients. Four of the five patients were analyzed both at relapse and in remission. Three of the four patients were first analyzed at relapse and followed-up at later time points. The increased frequency of CD56^+^ T cells slightly decreased in all three patients 3 months after relapse (Fig. 7a).

One of the three patients suffered a second relapse 13 months after the first relapse (presented as squares in Fig. 7). The frequency of CD56^+^ T cells in this patient decreased from 68.3% at the first relapse to 15.9% at 7 months later, but increased again to 56.6% in the second relapse. We were able to follow only one patient before the first relapse (presented as circles in Fig. 7). The frequency of CD56^+^ T cells 6 months before relapse was 22.2%, which was higher than that in the relapse-free F-MS patients. One patient, who experienced five relapses in 2 years while on fingolimod, was followed up at 3 months after the last relapse; 52.2% of this patient’s T cells were positive for CD56 (presented as a diamond in Fig. 7).

The mean frequency of granzyme B-expressing cells and IFN-γ-producing cells in the CD56^+^ and CD56^−^ T cell subsets decreased in each patient 3 months after relapse (Fig. 7b). The frequency of CD56^+^ T cells in the relapse-experienced F-MS patients was significantly higher even during remission than that in the relapse-free F-MS patients (34.4% vs. 10.8%, p = 0.0013) (Fig. 7c).

### Fingolimod therapy does not affect the frequency of Foxp3^+^ regulatory T cells

**Figure 8:**
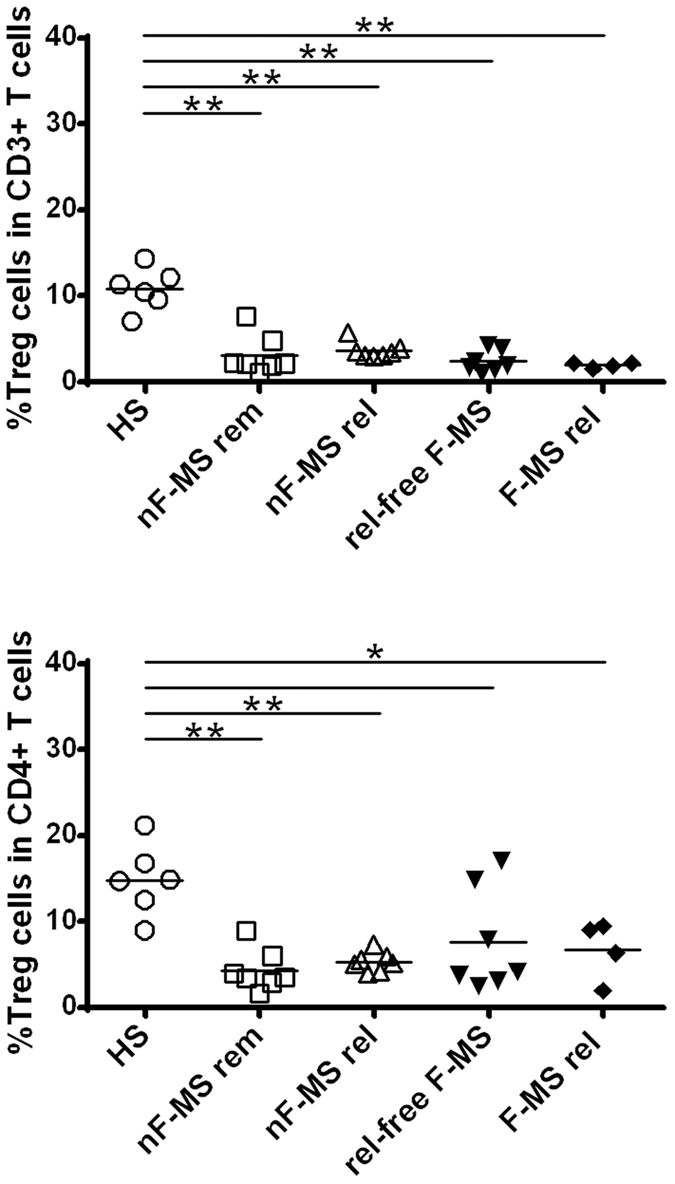
The frequency of Foxp3+ regulatory T cells in multiple sclerosis patients and healthy subjects. The frequency of regulatory T cells (Treg cells: CD3^+^CD4^+^CD25^+^Foxp3^+^ cells) in CD3^+^ T cells (upper) and CD4^+^ T cells (bottom). HS: healthy subjects, nF-MS: multiple sclerosis patients without fingolimod therapy, F-MS: multiple sclerosis patients with fingolimod therapy, rem: remission, rel: relapse, rel-free: relapse-free. Bars represent median values. p-values: *p < 0.05; **p < 0.01.

Next, we evaluated the effect of fingolimod therapy on Foxp3^+^ regulatory T cells (Foxp3^+^ Treg). The frequency of Foxp3^+^ Treg decreased significantly in nF-MS patients, both during relapse and while in remission, compared with HS (p = 0.0023 and p = 0.0012, respectively) (Fig. 8). Similar to nF-MS patients, both relapse-free and relapsed F-MS patients showed significantly lower levels of Foxp3^+^ Treg compared with HS (p = 0.0095 and p = 0.0012, respectively). There was no significant difference in the frequency of Foxp3^+^ Treg between nF-MS and F-MS patients. These results indicate that fingolimod therapy did not have a large effect on the frequency of Foxp3^+^ Treg.

## Discussion

The first striking finding in this study is that the frequency of CD56^+^ T cells increased in fingolimod-treated patients, which was not observed in patients without MS-DMD and IFN-β therapy. Notably, these T cell populations appeared to be related to relapse under fingolimod therapy because they further increased during relapse. In contrast, CCR7^+^ TCM rather decreased in relapsed F-MS patients more than those in relapse-free F-MS patients, indicating that an insufficient reduction in TCM in the peripheral blood cannot fully explain relapse on fingolimod in this study.

CD56, an isoform of the neural cell adhesion molecule, is expressed by neural and glial cells in the CNS and by NK cells and some T cells^23^,^24^. Although the role of CD56 on immune cells has not been clearly determined, CD56^+^ immune cells are considered to be cytotoxic, and the homotypic interaction with CD56 may facilitate the cytotoxicity toward target cells^24^. There are a few reports on CD56^+^ T cells in MS patients. CD4^+^CD56^+^ MBP-specific T cells are cytotoxic to MBP-pulsed target cells in an MHC class II-restricted manner, and they can lyse oligodendrocytes via a mechanism that is independent of MHC class II restriction^18^,^19^,^25^. However, the association of CD56^+^ T cells with MS pathogenesis has not been widely recognized likely because, as shown here, CD56^+^ T cells were not increased in patients who did not receive fingolimod therapy, regardless of remission or relapse, and most of the CD56^+^ T cells were CCR7-negative, despite that encephalitogenic cells are thought to belong to TCM expressing CCR7^12^,^26^.

Fingolimod therapy may place MS patients in different immune situations although fingolimod itself does not necessarily exert a direct effect on upregulation of CD56 on T cells. Fingolimod has some direct effects on immune cells mediated by S1P1, but may rather indirectly change immune system by unidentified mechanisms. Thus, relapses while on fingolimod therapy may be due to different pathological mechanisms from ordinary MS relapses. It is difficult to determine the role of CD56^+^ cells within the circulating T cell population in relapse of fingolimod-treated patients. Cytotoxicity by CD56^+^ T cells may be associated with relapse on fingolimod because CD56^+^ T cells express various molecules that are capable of exerting both antigen-specific and MHC-unrestricted cytotoxicity^22^,^25^,^27^,^28^. In this regard, the expression of cytotoxic molecules may be more essential than CD56 because the upregulation of granzyme B, perforin, and FasL was greater in patients under fingolimod therapy, particularly during relapse, and these molecules also increased in CD56^−^ T cells.

We also expected that the analysis of cytokine production by CD56^+^ T cells and CD56^−^ T cells would provide information regarding another possible pathomechanism of relapse on fingolimod because IFN-γ-producing cells and IL-17-producing T cells are considered to play a central role in the pathogenesis of MS^29^,^30^,^31^,^32^. The frequency of IFN-γ-producing cells increased in fingolimod-treated patients, both in response to PMA/ionomycin and to MBP. IFN-γ-producing cells increased in CD56^−^ T cells, as well as CD56^+^ T cells derived from fingolimod-treated patients particularly during relapse. However, the antigen-reactive response to MBP was significantly higher in CD56^+^ T cells than in CD56^−^ T cells. Although the frequencies of IL-17A-producing cells increased at relapse in some of the nF-MS patients, IL-17A-producing cells did not increase during relapse on fingolimod.

We considered that the induction of IFN-γ bias by fingolimod therapy occurred both in CD56^+^ T cells and in CD56^−^ T cells regardless of antigen reactivity and that CD56^+^ T cells may contain a greater number of pathogenic T cells. Thus, it is likely that CD56^+^ T cells contribute to relapse in fingolimod-treated patients by producing IFN-γ independently of cytotoxicity towards neural cells via various cytotoxic molecules. On the other hand, the roles of IL-17A-producing cells must be carefully interpreted. We ensure that IL17A-bias did not occur in our patients in comparison to IFN-γ. But further studies are needed to unravel roles of IL-17A-producing cells in MS following fingolimod treatment.

One mechanism that may increase the frequency of CD56^+^ T cells is the decrease of CCR7^+^ cells by fingolimod. However, other mechanisms should be considered because CD56^+^ T cells increased even within CCR7^+^ T cells and increased frequency of CD56^+^ T cells was extended to all T cell populations in fingolimod-treated patients. It is unlikely that fingolimod or fingolimod-phosphate directly induced CD56 expression because we cultured PBMC with fingolimod or fingolimod-phosphate, which failed to induce CD56 on T cells. We also evaluated whether fingolimod further upregulated CD56 on T cells when co-cultured with PHA. As reported previously^25^, culture with PHA exhibited effects to upregulate CD56, but addition of fingolimod or fingolimod-phosphate did not change the degree of CD56 upregulation. Therefore, other causes of CD56 upregulation rather than direct effects of fingolimod on immune cells should be investigated. We also confirmed that molecules contained by fingolimod-exposed serum were not sufficient to upregulate CD56 on T cells *in vitro*.

The upregulation of CD56 on T cells was observed under various conditions *in vivo*, such as infection, autoimmune diseases, and cancer^33^,^34^,^35^,^36^,^37^. Stimulation of PBMC or T cells with IFN-γ, anti-CD3, and IL-2 or PHA generates CD56^+^ T cells mainly from CD56^−^ T-cell progenitors *in vitro*^25^,^28^,^38^. *In vitro*-established CD56^+^ T cells have natural killer (NK) cell-like cytotoxic activity and have been used for the immunotherapy of various tumors^39^,^40^. However, circulating CD56^+^ T cells exhibit different characteristics from *in vitro*-generated CD56^+^ T cells^28^. The origin and function of circulating CD56^+^ T cells remains elusive. In our study, we observed an elevated frequency of CD56^+^ T cells in the peripheral blood of fingolimod-treated MS patients. Our patients did not have malignancy, CD56 upregulation was not consistently reported in autoimmune diseases, including nF-MS patients in this study^35^,^41^,^42^,^43^. Thus, the association between infectious pathogens and CD56 upregulation is of particular interest. CD56^+^ T cells were reported to play important roles in preventing viral replication through IFN-γ production^44^,^45^. The upregulation of CD56 on T cells was observed even in healthy cytomegalovirus-positive subjects^46^. Fingolimod therapy may increase virus quantities *in vivo;* fingolimod therapy allowed for the expansion of Epstein-Barr virus to sufficient level to detect viral DNA in the saliva^47^. Moreover, it was recently reported that fingolimod worsen the severity of viral-induced demyelination^48^. Collectively, we hypothesize that the CD56 upregulation observed in our study was a response to increased virus quantities under fingolimod therapy, although further studies are needed to confirm this hypothesis.

To evaluate whether a lower number of regulatory cells is associated with relapses, we examined the frequency of Foxp3^+^ Treg. We confirmed decreased frequency of Treg in MS, which was also reported previously^49^,^50^,^51^. The frequencies of Foxp3^+^ Treg did not significantly vary between fingolimod-treated and -untreated patients. However, our analysis was still limited and further studies are necessary to confirm our observation.

One of the clinically noteworthy findings of this study is that the CD56^+^ population was increased 6 months before relapse in one patient. Relapse-experienced F-MS patients retained a high frequency of CD56^+^ T cells even during remission. Our data has raised a possibility that the frequency of CD56^+^ T cells is a useful biomarker for predicting relapses, although further confirmation of this measure is required. As discussed above, the upregulation of the cytotoxic molecules and IFN-γ production by T cells was observed in CD56^−^ T cells, as well as CD56^+^ T cells, which may be more functionally relevant to relapses than the upregulation of CD56. However, CD56 is a surface marker, which may have greater advantage as a potential biomarker in the clinical setting.

In summary, we found that cytotoxic T cells that tended to express CD56 were increased in fingolimod-treated patients, particularly during relapses. These T cells showed a Th1-like response, and a considerable number of the CD56^+^ T cells reacted with MBP. We speculate that CD56^+^ T cells exhibits an autoaggressive role via cytotoxicity and/or IFN-γ production and play a crucial role in relapses as cryptic encephalitogenic T cells under fingolimod therapy.

## Methods

### Patients and healthy individuals

A total of 33 patients with relapsing-remitting MS and 10 HS were enrolled in this study. All MS patients met the McDonald’s criteria and were negative for the aquaporin-4 antibody. Fingolimod was administered to 16 patients (F-MS). Of these, 11 F-MS patients did not experience relapses (relapse-free F-MS), whereas five experienced relapse (relapse-experienced F-MS). Relapse samples were available from four of the five relapse-experienced F-MS (relapsed F-MS). Twelve patients received IFN-β therapy, and eight patients did not receive any DMD at the time of first specimen collection. One patient switched treatment from IFN-β to fingolimod, and two patients started treatment with fingolimod. Sampling was performed multiple times in some patients. When therapies were switched, patients were permitted to be added to more than one group based on the therapy at the time of sampling. In our study, “in remission” was defined as a clinically stable state of more than 30 days. “At relapse” was defined as the period within 14 days after the initiation of neurological exacerbation; this “exacerbation” indicates neurological episodes lasting more than 24 hours. Characteristics of the enrolled MS patients and HS are listed in Table 1.

All experimental protocols of the present study were approved by the Medical Ethics Committee of Kyoto Prefectural University of Medicine (RBMR-G-140-1), and all subjects provided written informed consent before they were enrolled in this study. The methods were carried out in accordance with the approved guidelines.

### Cell preparation and sorting

PBMC were isolated through gradient centrifugation with Lymphocyte Separation Medium 1077 (Wako, Osaka, Japan), according to the manufacturer’s instructions. Purified CD56^+^ and CD56^−^ T cells were isolated using a Cell Sorter SH800 (Sony, Tokyo, Japan), using anti-CD3 monoclonal antibody (mAb) and anti-CD56 mAb, and were confirmed to be ≥95% pure.

### Flow cytometric analysis of T cell surface and intracellular proteins

PBMC were stained with anti-human mAbs conjugated with fluorochromes for 20 minutes in the dark at 4 °C and washed twice with Dulbecco’s phosphate-buffered saline containing 2% fetal bovine serum (FBS). The following mAbs were used for surface phenotypic analyses: anti-CD3 fluorescein isothiocyanate (FITC) (clone UCHT1), anti-CD4 peridinin chlorophyll protein-Cy5.5 (PerCP-Cy5.5) (clone SK3), anti-CD8 phycoerythrin (PE), anti-CD8 phycoerythrin-Cy7 (PE-Cy7), anti-CD8 allophycocyanin-Cy7 (APC-Cy7) (all clone SK1), anti-CD25 PE (clone M-A251), anti-CD45RA APC-H7 (clone HI100), anti-CD56 APC, anti-CD56 PE (both clone B159), anti-CCR7 PE-Cy7 (clone 3D12), (all from BD Biosciences, Franklin Lakes, NJ, USA), anti-Fas ligand PE (clone NOK-1, BioLegend, San Diego, CA, USA), and isotype-matched control monoclonal antibodies (all from BD Biosciences). After staining the cell surfaces for CD56, CD3, CD4, and CD8, the cells were fixed with intracellular fixation buffer and intracellular permeabilization buffer (eBioscience, San Diego, CA, USA) and then stained with anti-granzyme B Alexa Fluor 647 (clone GB11, BD Biosciences) and anti-perforin APC (clone dG9, eBioscience). To analyze Foxp3 expression to detect regulatory T cells, the cells were fixed and permeabilized with human FoxP3 buffer set (BD Biosciences) after surface staining for CD3, CD4, and CD25 and then stained with anti-Foxp3 Alexa Fluor 647 (clone 259D/C7, BD Biosciences). Isotype-matched control monoclonal antibodies were used for all staining. All flow cytometric data were acquired on a FACS Canto II flow cytometer (BD Biosciences) and analyzed with FlowJo software (TreeStar, Ashland, OR, USA).

### Flow cytometric analysis of cytokine production by T cells stimulated with PMA/ionomycin

To analyze the frequency of cytokine-producing cells, PBMC suspended in AIM-V (Thermo Fisher Scientific, Walthman, MA, US) at 10^6^ ml^−1^ in 96-well U-bottom plates were stimulated with Cell Stimulation Cocktail (eBioscience), which included PMA/ionomycin. PBMC were stimulated with the cocktail in the presence of monensin (eBioscience) for 4 hours before staining, according to the manufacturer’s instructions. The stimulated cells were surface-stained for CD56, CD3, CD4, and CD8, fixed with intracellular fixation buffer and intracellular permeabilization buffer (eBioscience), and then stained with anti-IFN-γ PE-Cy7 (clone B27) and anti- interleukin (IL)-17A PE (clone N49-653) (all from BD Biosciences). Appropriate isotype control reagents were used in each experiment. Data were acquired on a FACS Canto II flow cytometer and analyzed using FlowJo software.

### *In vitro* analysis of effect of fingolimod regarding CD56 expression on T cells

PBMC from healthy subjects and MS patients without DMD were cultured for 5 days at 1 × 10^6^ cells/ml in the presence of fingolimod (0.01, 0.1, 1, 5, 10 μM; Cayman Chemical, Ann Arbor, MI,USA) or fingolimod-phosphate (0.01 μM, 0.1 μM, 1 μM, 5 μM, 10 μM; Cayman Chemical) in AIM-V medium. On day 3 and day 5, CD56 expression was analyzed as described above.

To upregulate CD56 on T cells, PBMC from three HS and three MS without DMD were also cultured for 5 days at 1 × 10^6^ cells/ml in the presence of PHA (2 mg/ml; Sigma-Aldrich, St. Louis, MO, USA) in AMI-V supplemented with 10% FBS. Fingolimod or fingolimod-phosphate was added to the cultured wells at the concentration of 0.1 μM and 1 μM, and compared to the culture with PHA alone.

### *In vitro* analysis of effect of the serum from F-MS patients regarding CD56 expression on T cells

PBMC were derived from three MS patients for whom fingolimod therapy was introduced during this study. PBMC derived when without DMD were cultured with various densities of autologous serum taken after the initiation of fingolimod therapy for 5 days at 1 × 10^6^ cells/ml. The serum was not inactivated, and an increased frequency of CD56 positive T cells over 10% was confirmed at serum sampling. CD56 was analyzed as described above.

### MBP-reactive proinflammatory cytokine production

Purified CD56^+^ and CD56^−^ T cells were obtained from five MS patients receiving fingolimod treatment, including three relapse-free and two relapse-experienced patients in remission. Sorted CD56^+^ and CD56^−^ T cell subsets (1 × 10^5^ cells/mL) were cocultured with mitomycin C-treated (Nacalai tesque, Kyoto, Japan) antigen-presenting cells (APCT) (2 × 10^5^ cells/mL), in the presence or absence of MBP peptide mixture (PepTivator MBP Isoform 5, MACS, Milteny Biotec, Bergisch, Germany) for 10 hours, according to the manufacturer’s instructions. These cells were treated with monensin for the final 6 hours of culture. The stimulated cells were surface-stained for CD3, CD4, and CD8 and then stained intracellularly for IFN-γ and IL-17A according to the protocols described above. Data were acquired on a FACS Canto II flow cytometer and analyzed with FlowJo software. MBP-reactive proinflammatory cytokine production was evaluated as a delta frequency that was calculated by subtraction of the frequency of cytokine-producing cells in the absence of MBP from that in the presence of MBP.

### Statistical analysis

Statistical analyses were performed using the Mann–Whitney U test and Wilcoxon signed-rank test for comparisons between the two groups of data. The paired t-test was used to analyze MBP-reactive cytokine production. All data sets were analyzed using GraphPad Prism software (GraphPad, San Diego, CA, USA). Values of p < 0.05 were considered to be significant.

## Additional Information

**How to cite this article**: Fujii, C. *et al*. Altered T cell phenotypes associated with clinical relapse of multiple sclerosis patients receiving fingolimod therapy. *Sci. Rep.* **6**, 35314; doi: 10.1038/srep35314 (2016).

## Figures and Tables

**Table 1:** Demographic characteristics of multiple sclerosis patients (MS) and healthy subjects (HS) enrolled in the study.

	HS	MS patients without fingolimod therapy				MS patients with fingolimod therapy		
		without DMD		with IFN-β		relapse-free	relapse-experienced	
No. of subjects	10	8		12		11	5	
Phases of sampling		5 in rem	5 at rel	11 in rem	4 at rel	11 in rem	5 in rem	4 at rel
Age (mean)	35.1	32.6	28.0	39.4	46.5	41.8	46.8	48.7
Gender (F:M)	7:3	5:0	4:1	8:3	3:1	7:4	4:1	3:1
Disease duration (years)		8.5	0	8.0	10.7	7.2	6.0	6.7
Duration of fingolimod therapy (year)						1.0	1.2	1.0

Sampling was performed multiple times in some patients. When therapies were switched, patients were allowed to be added to more than one group based on the therapy at the time of sampling. Similarly, when samplings were performed both in remission and at relapse, the patients were added to both groups in remission and at relapse. Fingolimod-treated patients are divided by whether or not relapses were experienced. Abbreviations are as follows, without DMD: without disease-modifying drug, with IFN-β: with interferon β therapy, rem: remission, rel: relapse.

**Table 2:** Absolute number of different T cells populations of multiple sclerosis patients (MS) with or without fingolimod therapy.

	MS patients without fingolimod therapy				MS patients with fingolimod therapy		
	without DMD		with IFN-β		relapse-free		relapse-experienced
	in rem	at rel	in rem	at rel	in rem	at rel	3 month after rel
No. of patients	5	5	11	4	11	4	4
Lymphocytes/μl	1548 ± 378.1	1284 ± 424.0	1437 ± 534.7	1795 ± 335.2	433.6 ± 196.9	425 ± 152.3	455 ± 151.7
*T cells/μl*							
Total	1281 ± 386	964.3 ± 312.9	1134.9 ± 431.4	1377.6 ± 344.6	162.8 ± 155.8	122.7 ± 45.9	117.7 ± 83.3
*CD56*+*cells/μl*							
CD3^+^	43.9 ± 24.0	16.6 ± 11.0	29.4 ± 34.0	14.7 ± 14.0	20.1 ± 23.0	71.1 ± 36.0	56.9 ± 43.9
CD4^+^	8.7 ± 4.0	6.3 ± 3.0	3.9 ± 2.0	6.3 ± 7.0	2.8 ± 3.0	10.9 ± 13.0	11.1 ± 11.6
CD8^+^	24.2 ± 15.0	8.8 ± 7.0	21.2 ± 27.0	8.1 ± 7.0	10.5 ± 15.0	40.8 ± 15.0	23.5 ± 10.3

Mean values (± standard deviation) are reported.

Abbreviations are as follows, without DMD: without disease-modifying drug, with IFN-β: with interferon β therapy, rem: remission, rel: relapse.
